# Molecular pathology provides significant insights into the diagnosis and treatment of cancer of unknown primary origin: a case report of an extremely rare male occult breast apocrine cancer

**DOI:** 10.1007/s13691-025-00840-4

**Published:** 2026-04-11

**Authors:** Yusuke Hayashi, Taichi Yoshida, Koji Fukuda, Kazuhiro Shimazu, Daiki Taguchi, Naoaki Kodama, Tomohiro Matsumoto, Anna Terasawa, Eriko Takahashi, Yuko Hiroshima, Kazuhiro Imai, Hiroshi Nanjyo, Hiroyuki Shibata

**Affiliations:** 1 Department of Clinical Oncology, Graduate School of Medicine, Akita University, Hondo1-1-1, Akita, 010-8543 Japan; 2 Department of Thoracic Surgery, Akita University Graduate School of Medicine, Hondo 1-1-1, 010-8543 Akita, Japan; 3 Department of Pathology, Akita University Hospital, Hondo1-1-1, Akita, 010-8543 Japan; 4 Center for Cancer Registry and Information Services, Akita University Hospital, Hondo 1-1-1, Akita, Japan

**Keywords:** Cancer of unknown primary, Immunohistochemistry, Occult male breast apocrine carcinoma

## Abstract

We present a case of occult apocrine breast cancer in a man presenting with back pain and multiple bone and large liver metastases. The primary tumour was not identified, and the patient was initially considered to have cancer of unknown primary origin. During referral to our hospital, 2 months had passed since the initial consultation, and the prognosis was considered poor. Empirical treatment with carboplatin and paclitaxel was then administered. Histological examination of the liver metastases revealed large cells with eosinophilic cytoplasm, morphologically suggestive of apocrine breast cancer, but no tumour was found in the breast. The diagnosis of occult apocrine breast cancer was confirmed by concurrent immunohistochemical examination of the biopsy tissue sample, revealing androgen receptor positivity, oestrogen receptor negativity, gross cystic disease fluid protein 15 positivity, human epidermal growth factor receptor 2 overexpression, and gene amplification. Carboplatin and paclitaxel treatment resulted in the near-complete remission of liver metastases and reduction of bone metastases. The reduction has still been maintained at 16 months since the symptom onset. Molecular targeted therapy, including trastuzumab, is expected to further extend survival. Male occult apocrine breast cancer is extremely rare, with only one other case reported in the literature. We report the diagnosis and treatment response of this extremely rare case.

## Introduction

Cancer of Unknown Primary (CUP) is a metastatic malignant tumour whose primary site cannot be identified even after an intensive examination. It accounts for approximately 2%–5% of all malignant tumours [1]. Given that the primary site is unknown, administering systemic chemotherapy based on the type of primary malignancy is challenging, and the median survival time is extremely poor, typically approximately 6–12 months [1, 2]. Conversely, in patients with a favourable prognosis, where the primary site is strongly suspected based on the histological type, clinical features, and immunohistochemical (IHC) staining results, long-term survival may be expected with specialized treatment [3].

In CUP management, basic blood tests and relevant tumour markers are examined, and computed tomography (CT) and positron emission tomography (PET)-CT are performed to identify unrecognized malignant lesions. Subsequently, the histological type of the metastatic lesions is confirmed, and IHC staining is used to aid in identifying the primary site [4]. Particularly, for adenocarcinomas, a combination of markers such as GATA binding protein 3 (GATA3), thyroid transcription factor-1 (TTF-1), and caudal type homeobox 2 (CDX2), in addition to the cytokeratin (CK) 7, and CK20 expression patterns, plays an important role in enhancing the diagnostic accuracy [4]. These results may facilitate the induction of organ-specific treatments, when possible, rather than empirical chemotherapy [5].

Breast cancer basically affects women, but it occasionally occurs in men. Male breast cancer (MBC) accounts for < 1% of all breast cancers, and occult male breast cancer (OMBC) cases are extremely rare [6]. When the results of the IHC analysis suggest a mammary origin, diagnostic clues include negativity for CK20 and positivity for CK7, GATA3, gross cystic disease fluid protein 15 (GCDFP15), and mammaglobin [6, 7]. Furthermore, human epidermal growth factor receptor 2 (HER2) overexpression or gene amplification may indicate the indications for molecular targeted therapy [8, 9].

Our case primarily presents a poorly differentiated adenocarcinoma with unknown primary and multiple liver and bone metastases. After detailed examination including IHC, an occult breast cancer was strongly suspected. To the best of our knowledge, only a total of 14 OMBC cases have reported for 17 years since 2008. We report another rare case, along with a review of the relevant literature, to aid in the diagnosis and treatment of this rare entity.

### Case report

**Fig. 1:**
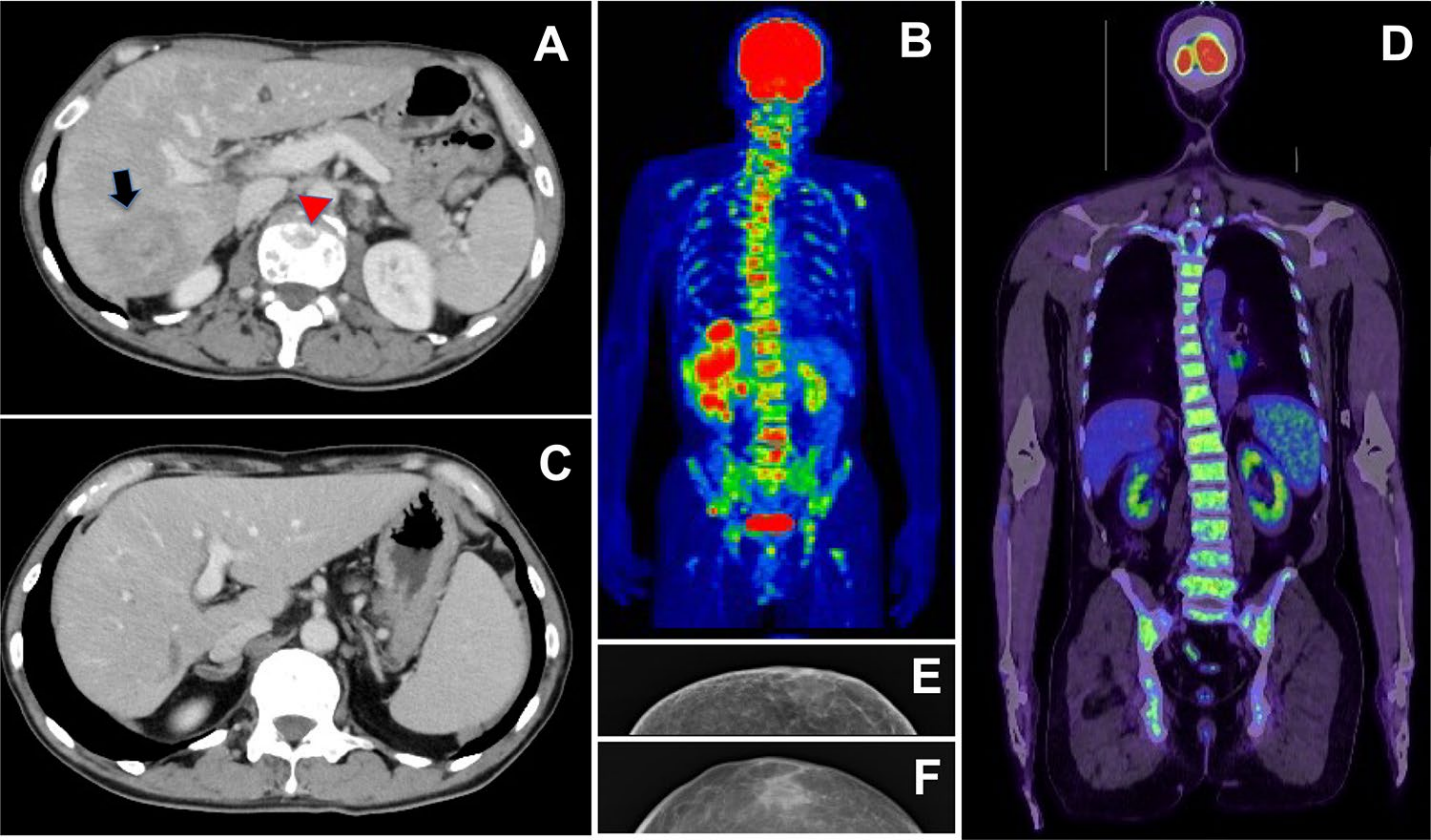
Patient images. **A**. CT image at the initial examination. The black arrow indicates liver metastasis, and the red arrowhead indicates bone metastases. **B**. PET-CT image before chemotherapy. The regions with higher uptake of 18FDG signal are indicated in the liver and whole spine. **C**. CT image after five cycles of CBDCA + PTX treatment. Liver metastasis disappeared and the spotty osteolytic region was repaired. **D**. PET-CT image after eight cycles of CBDCA + PTX treatment. The uptake in the liver disappeared and the spine signals decreased. **E**. Mammography of the right breast. **F**. Mammography of the left breast. No tumour was detected in both breasts

**Fig. 2:**
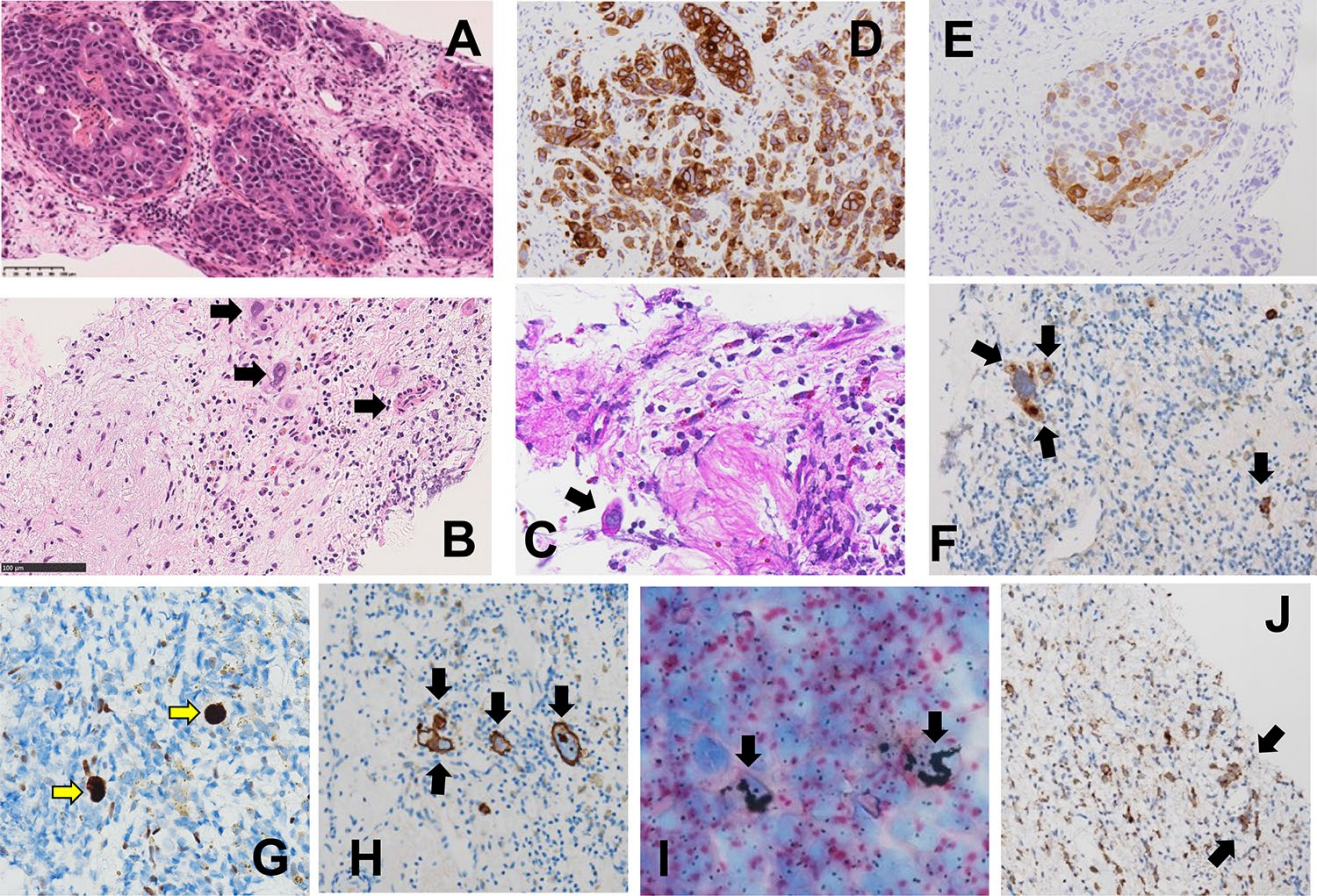
Histology and immunohistochemistry of the liver tumour. **A**. Haematoxylin and eosin (HE) staining. **B**. HE staining of the second biopsy after treatment. The black arrows indicate the residual tumour cells. **C**. PAS staining of the residual tumour cell. **D**. Immunohistochemistry (IHC) of the original liver tumour with anti-CK 7 antibody. **E**. IHC of the original liver tumour with anti-mammaglobin antibody (higher magnification). **F**. IHC of the residual tumour cells with anti-GCDFP15 antibody (higher magnification). **G**. IHC of the residual tumour cells with anti-androgen receptor antibody (higher magnification). The yellow arrows indicate the residual tumour cells. **H**. IHC of the residual tumour cells with anti-HER 2 antibody. The black arrows indicate the residual tumour cells. **I**. HER2 DISH of the residual tumour cells. The black bundle indicates HER2 gene amplification corresponding to 12 copies. The black dot indicates HER2 gene in the surrounding stromal cells. The pink dot indicates the centromere of chromosome 17. The black arrows indicate the residual tumour cells. **J**. IHC of the residual tumour cells with anti-PD-L1 antibody. The black arrows indicate the residual tumour cells

A 56-year-old man visiting Hospital X in July 2024 complained of lower back and bilateral leg pains. At the initial visit, blood tests revealed mild elevations in liver enzymes, and contrast-enhanced CT scans showed multiple mass lesions centred on the liver S6 region (Fig. 1A). Additionally, bone scintigraphy and PET-CT confirmed multiple bone metastases, involving nearly the whole spine (Fig. 1B). A percutaneous biopsy of the liver tumour revealed a pathological diagnosis of a poorly differentiated adenocarcinoma (Fig. 2A), with IHC positivity for CK7 (Fig. 2B) and negativity for CK20. Given that the primary tumour was not identified at this time, he was referred to our department in September of the same year for the evaluation of CUP [1, 4].

Although this diagnosis was later proven to be incorrect, the patient was initially diagnosed with CUP with multiple liver and bone metastases, and the prognosis at that time was considered to be poor. As the patient was living alone, his previous doctor advised him to return to his hometown where his relatives resided, and he was subsequently referred to our hospital.

At the initial visit in our hospital, the patient’s performance status was 1, he had mild weight loss (7 kg in 6 months), and his tumour markers were elevated (carcinoembryonic antigen: 94 ng/mL, cancer antigen 15–3: 305.6 ng/mL, carbohydrate antigen 19–9: 24.4 U/mL), but his PSA was within the normal range at 0.63 ng/mL, ruling out prostate cancer. CT and PET-CT scan revealed multiple tumours measuring < 6 cm in the liver, as well as multiple bone metastases to the spine through the cervical (C2) to the lumbar regions (L5) (Fig. 1A, B).

The current Japanese guidelines recommend that CUP treatment should be initiated within 1 month of diagnosis [5], but in the present case, nearly 2 months had passed since the initial consultation before treatment began. Owing to the difficulties in identifying the primary organ based on the pathological histology and rapid clinical progression, empirical chemotherapy with carboplatin (CBDCA, AUC = 3.5) and paclitaxel (PTX, 120 mg/m2) was selected [5, 8]. At 7 days after the initial administration, he developed grade 4 febrile neutropenia. The next day, he was diagnosed with sepsis and hospitalized. However, he recovered after treatment with granulocyte-colony stimulating factor (G-CSF) and carbapenem antibiotics. From the second treatment course onwards, PTX was continued at a 70% dose reduction. Treatment was performed approximately once a month, and polyethylene glycol-G-CSF was used in combination from the third treatment. To date, 13 treatment courses have been continued. Furthermore, the bone-modifying drug, denosumab, was administered every 4 weeks to treat bone metastases.

CT performed at the end of the third treatment course showed a significant reduction in liver metastases, with imaging findings suggesting a near-complete response (Fig. 2C). A PET-CT scan performed in April 2025 showed that the liver uptake had almost disappeared, the widespread spinal uptake had also been reduced to a spotty state, and bone metastasis appeared to be under control (Fig. 2D).

A subsequent detailed pathological examination revealed a poorly differentiated adenocarcinoma with cord-like, acinar-like, and tubular invasive proliferation of medium-to-large-sized adenocarcinoma cells with enlarged nuclei, condensed nuclear chromatin, and weakly eosinophilic cytoplasm, accompanied with fibrous stroma and inflammatory cell infiltration of lymphocytes, leading to a diagnosis of apocrine carcinoma (Fig. 2A). The pretreatment biopsy tissue was entirely consumed by IHC. An additional liver biopsy performed after chemotherapy revealed a considerable decrease in the tumour cell number (Fig. 2B). Periodic acid-Schiff stain (PAS) staining showed positive findings (Fig. 2C).

The IHC analyses revealed positivity for CK7 (Fig. 2D), mammaglobin (Fig. 2E), GATA3, GCDFP15 (Fig. 2F), and androgen receptor (AR) (Fig. 2G) and negativity for CK20. Furthermore, the examination revealed negativity for estrogen receptor (ER), progesterone receptor (PgR), thyroglobulin, TTF-1, paired box 8 (PAX8), p40, p63, CDX-2, CK5/6, and anti-hepatocyte specific antigen. The few remaining tumour cells in the additional liver biopsy showed strong HER2 3 + positivity (Fig. 2H), and HER2 gene amplification was confirmed by DISH (gene amplification: > 12, Fig. 2I) [9]. Based on the above-mentioned findings, our case presents an extremely rare occurrence of occult male HER2-positive breast cancer.

Programmed cell death 1 (PD-1) and programmed death-ligand 1 (PD-L1) were also positive (Fig. 2J). The patient’s combined positive score (CPS) was 30. All mismatch repair proteins were positive, indicating no mismatch repair deficiency. The IHC results are summarized in Table 1. These results suggested an occult male breast apocrine carcinoma (OMBAC). Additional mammography and breast ultrasound examination revealed no obvious abnormalities (Fig. 1E, F).

**Table 1:** Summary of the IHC staining results

Marker		Marker		Marker	
CK 7	+	AR	+	Thyroglobulin	-
GATA3	+	PD-1	+	TTF-1	-
Mammaglobin	±	PD-L1	+	SMRCA4/BRG1	+
HER2	3 +	(CPS)	30	PAX 8	-
GCDFP15	+	MLH1	+	p40	-
CK20	-	MSH2	+	p63	-
ER	-	MSH6	+	CDX-2	-
PgR	-	PMS2	+	Ki-67	< 1%

Antibodies (Abs) used in this case report were anti-CK7 Ab (SP52), anti-GATA3 Ab (L50-823), anti-mammaglobin Ab (31A5), anti-HER2 Ab (4B5), anti-GCDFP-15 Ab (EP1582Y), anti- CK20 Ab (SP33), anti-ER Ab (SP1), anti-PgR Ab (clone 1E2), anti-AR Ab (SP107), anti- PD-1 Ab (NAT105), anti-MLH1 Ab (M1), anti-MSH2 Ab (G21901129), anti-MSH6 Ab (SP93), anti-PMS2 Ab (A16-4), anti-TTF-1 Ab (TSSC3), anti-PAX8 Ab (MRQ-50), anti- p40 Ab (BC28) and anti-p63 Ab (4A4) were purchased from F. Hoffmann-La Roche, Ltd. (Basel, Switzerland). anti-PD-L1 Ab (clone 28–8) and anti-SMACA4/BRG1 Ab (EPNCIR111A) were purchased from abcam (Cambridge, UK). Anti-thyroglobulin Ab (8G7G3/1) and anti-Ki-67 Ab (MIB-1) from Agilent Technologies, Inc. (Santa Clara, California, U.S.), and anti-Ki-67 Ab (CDX2-88) from BioCare (Taoyuan City, Taiwan).

Following 1 year and 1 month after treatment initiation, no liver metastasis has been detected, bone metastasis remains under control, and treatment with CBDCA + PTX is still effective. If the patient’s condition deteriorates, anti-HER2 therapy (e.g. trastuzumab and pertuzumab) will be considered. Owing to the patient’s low cell count, cancer genome panel testing was not performed.

## Discussion

We report a case of adenocarcinoma of unknown primary site with multiple metastases to the liver and bone.

MBC accounts for approximately 0.7% of all breast cancers worldwide [10]. OMBC was identified in a very small fraction (0.13%) of MBC patients (n = 23,374) in a previous report [11]. A simple calculation suggests that OMBC accounts for 0.09% of breast cancers. The diagnostic rate of OMBC is very low; thus, little is known about this disease. To the best of our knowledge, only 15 cases have been reported in the literature [12]. In the 14 cases reported, the initial presentation was an axillary mass; in one case, the tumour was located on the anterior chest wall (Table 2). Two thirds of the OMBC cases (67%) were adenocarcinomas. Apocrine carcinoma, an atypical type of breast cancer, was identified in only one case (7%). Bone metastases were also rarely observed, occurring in only two cases (13%). In nearly all OMBC cases, the initial manifestation was axillary lymph node metastasis (Table 2) [13, 14].

**Table 2:** Characteristics of occult male breast cancer

Occult male breast cancer	Historical cases (ref. 12 and 14) 15 cases (2008–2025)	Our case
Median onset age (range)	58 years old(29–84)	
Tumor location	Axillary region (Including axillary LN): 14 (93%) Bone metastases: 2(13%) Chest wall: 1(7%)	Bone: multiple Liver
Pathological findings	Invasive ductal carcinoma:3(20%) Adenocarcinoma: 10 (67%) Lobular carcinoma: 1 (7%) Apocrine carcinoma: 1 (7%)	Apocrine carcinoma
Immunoreactivity	ER (+): 9 (60%) PgR (+):9 (60%) HER2 (+ ~ 3 +): 7 (7/14, 50%, NA in 1 case) dMMR: 0 (0%)	ER (-) PgR (-) HER2 (3 +) dMMR
Surgical treatment	Tumor resection: 11 (73%) Mastectomy: 6 (43%)	Not applicable
Chemotherapy	Trastuzumab: 4 (31%) Anthracycline: 6 (46%) Taxan: 8 (8/14, 57%, NA in 1 case) Cyclophosphamide: 3 (23%) Tamoxifen: 6 (6/14, 43%, NA in 1 case)	Paclitaxel + Carboplatine
Therapeutic outcomes	Disappearance (Including surgery): 9 (69%) SD: 2 (15%)	CR ~ PR

NA: not available

However, our case is unusual, as no axillary lesion was present and the disease primarily manifested as bone metastasis. Furthermore, our case was an apocrine carcinoma (Table 2).

The present case highlights the usefulness of IHC staining for estimating the primary site and determining the treatment options. IHC staining strongly suggested a breast cancer origin in our case [4, 6, 7].

The NCCN guidelines for CUP list the following IHC tests to predict breast cancer: CK7 +/CK20-, GATA3 +, GCDFP15 ±, mammagloblin ±, and ER/PR ± [8]. In diagnosing metastatic breast cancer, CK7 and CK20 are the most common IHC markers, and the differential diagnostic scope can be narrowed based on these markers. In breast cancer, CK7 positivity and CK20 negativity are common findings. CK7 is expressed in > 90% of breast cancer cases, whereas CK20 is mainly expressed in the gastrointestinal tumours [15].

Another IHC marker of great importance is GCDFP15, which has a positive predictive value and a specificity of 90% in diagnosing breast cancer [16, 17].

Apocrine carcinoma of the breast accounts for approximately 1% of all breast cancers [18]. Breast apocrine carcinoma refers to breast cancer cells resembling apocrine metaplastic cells [19].

OMBAC is estimated to account for 0.09% of breast cancers. The new 2019 World Health Organization defines apocrine carcinomas as follows [18]. Morphologically, they have abundant, granular, eosinophilic cytoplasm with well-defined borders and large nuclei with prominent nucleoli. They also show negativity for ER and positivity for cytokeratin, GCDFP15 and AR. In 30%–60% of apocrine carcinomas, HER2 positivity is present. They are also positive for GATA3 and α-methylacyl-CoA racemase.

GCDFP15 is a glycoprotein with a molecular weight of 15,000 and it was originally detected in the fluid of breast cysts. It is produced in normal tissues, such as the apocrine and salivary glands, as well as in the apocrine pseudo-epithelium of the mammary gland [20, 21]. Mazoujian reported that GCDFP15 corresponds to the intracellular vesicles and granules, which is believed to more accurately reflect the true apocrine characteristics [20].

The results of our IHC analysis were consistent with the above-mentioned molecular subtypes and was considered sufficient to diagnose OMBAC (Table 1). We believe that the clinicopathological features and treatment response of our rare OMBAC case, along with its molecular pathological characteristics, are worth reporting.

CBDCA + PTX were selected as empirical chemotherapy [5, 8, 22]. After three treatment courses, the patient’s liver metastases had almost completely disappeared, and bone metastases were reduced. Treatment with CBDCA + PTX reportedly has marked activity in advanced breast cancers and can be administered on an outpatient basis with manageable toxicity [22]. In our OMBC case, CBDCA + PTX was effective.

HER2 positivity confirmed by biopsy conducted after chemotherapy allowed the introduction of molecular targeted therapy [9]. Additionally, anti-PD-1/PD-L1 antibody might be effective for this OMBAC, because the CPS number was as high as 30. In cases of CUP where the primary site can be estimated, targeted therapy options are expanded, offering a potential for an improved prognosis. The poor prognosis of MOBC is attributed to its diagnosis at a late stage of disease progression [23, 24]. According to Parisi, MOBC may have axillary lymph node involvement, and the treatment outcomes are poor in advanced cases [6].

Chemotherapy for apocrine carcinoma is similar to that for general breast cancer. Conflicting results have been found in the literature regarding the response to chemotherapy and clinical outcomes of patients with apocrine breast cancer, which has been considered to have a poor prognosis. Conversely, approximately one-third of these cases have a better prognosis as compared to typical triple-negative breast cancer [18]. Given the HER2-positive status of our case, molecular targeted therapy was possible, providing important clinical implications for the treatment strategies for CUP.

The importance of careful histological examination and IHC staining is particularly emphasized in the treatment of OBC. This case underscores the importance of IHC staining for determining the primary tumour site and the potential for personalized treatment for HER2-positive tumour.

## Conclusion

Initially, our case was diagnosed as CUP, which is associated with a poor prognosis. However, microscopic imaging findings showed characteristics of an apocrine carcinoma and molecular pathological analysis confirmed the diagnosis of apocrine breast cancer with identifiable molecular targets, including HER2 and PD-1. Therefore, CUP must be diagnosed accurately through proper IHC analysis to ensure that potential good responders are not overlooked.

## Data Availability

Raw data were generated at Akita university. Derived data supporting the findings of this study are available from the corresponding author (HS) on request.
